# Edge fracture of thixotropic elastoviscoplastic liquid bridges

**DOI:** 10.1093/pnasnexus/pgad042

**Published:** 2023-02-09

**Authors:** San To Chan, Stylianos Varchanis, Amy Q Shen, Simon J Haward

**Affiliations:** Okinawa Institute of Science and Technology Graduate University, Onna, Okinawa 904-0495, Japan; Okinawa Institute of Science and Technology Graduate University, Onna, Okinawa 904-0495, Japan; Okinawa Institute of Science and Technology Graduate University, Onna, Okinawa 904-0495, Japan; Okinawa Institute of Science and Technology Graduate University, Onna, Okinawa 904-0495, Japan

**Keywords:** edge fracture, thixotropy, rheology, flow instability

## Abstract

It has recently been shown that torsion can break liquid bridges of viscoelastic fluids, with potential application to their clean and rapid dispensing. However, many commonplace fluids (paints, adhesives, pastes, and foodstuffs like chocolate) have more complex thixotropic elastoviscoplastic (TEVP) properties that depend on the imposed stress and the timescale of deformation. Using a commercial thermal paste, we show that liquid bridges of TEVP fluids can also be broken by torsion, demonstrating the applicability of the technique for improved dispensing of real industrial fluids. The liquid bridge breaking mechanism is an elastic instability known as “edge fracture.” Dimensional analysis predicts that the effects of thixotropy and plasticity can be neglected during edge fracture. Simulation using a nonlinear, phenomenological TEVP constitutive model confirms such a prediction. Our work yields new insight into the free-surface flows of TEVP fluids, which may be important to processes such as electronic packaging, additive manufacturing, and food engineering.

**Significance Statement**

Thixotropic elastoviscoplastic fluids such as adhesives can store energy elastically and resist extensional deformation. Consequently, a long ligament can form between the nozzle and the substrate when those fluids are dispensed, which may collapse after pinch-off and contaminate the underlying substrate. To overcome this stringing problem, we propose using edge fracture, the sudden indentation of the free surface of an elastic fluid subjected to sufficient torsion. Edge fracture cuts the fluid ligament horizontally without needing to apply any extension. Our work pushes the study of edge fracture, a flow instability that researchers tend to avoid in their experiments, into a new direction by showing how this “undesirable” phenomenon can be harnessed to benefit technological applications.

## Introduction

### Printability of fluids and the stringing problem

Modern dispensing technology such as direct ink writing (1–3) and fused deposition modeling (3–5) has driven myriad advances in areas like electronics, healthcare, robotics, and materials engineering. For electronics, the ability to print conductive and dielectric materials onto virtually any substrate has made the rapid prototyping and fabrication of new batteries (6–9), supercapacitors (10–13), sensors (14–17), and enclosures (18–21) possible. In the healthcare sector, polymeric, metallic, ceramic, and even living materials can be printed in nearly any shape and dimension, allowing the production of novel tissue scaffolds (22–25) and biological implants (26–29). The same is true for robotics, for which hydrogels, elastomers, and shape memory polymers can be printed and assembled into soft robots and actuators (30–33). For materials engineering, the ability to print porous, cellular, and other artificial structures have enabled the fabrication of meta-materials with extraordinary mechanical properties, such as ultralow density (34–36), tunable Poisson’s ratio (37), and negative stiffness (38). Nonetheless, the aforementioned technological achievements would have been impossible without considering the printability of the dispensed fluids.

For a fluid to be printable, it must demonstrate at least two physical properties. First, the fluid must be able to wet the dispensing substrate so that the printed structure stays stationary as the dispensing nozzle moves. Second, the fluid must have a viscosity that changes in response to stimuli such as the shear rate or temperature. Such a property helps the fluid flow more easily through the dispensing nozzle. More importantly, it enables the dispensed fluid to increase viscosity over orders of magnitude. Consequently, the printed structure can retain shape under its weight over a longer time and be solidified later by cooling, solvent evaporation, chemical cross-linking, or sintering (1, 3).

Unfortunately, the physical properties that render a fluid printable often make the dispensing process inefficient. Because the dispensed fluid tends to resist deformation, as the nozzle retracts, a relatively stable liquid bridge can form between the nozzle and the substrate, whose breakup can significantly slow down the dispensing process. To speed up the process, a common approach is to retract the nozzle over a longer distance and with a higher speed such that there will be more significant capillary and shear stresses to destabilize the liquid bridge. Yet, this reduces the precision as a long capillary tail can form after the liquid bridge pinches off. The capillary tail may then fall randomly onto and contaminate the dispensing substrate or the printed structure. This is often referred to as the stringing (or tailing) problem (20, 21, 39).

For particulate composites (fluids composed of particles suspended in a matrix) like conductive adhesives, surface-mount adhesives, and solder pastes, the stringing problem can be severe due to their material composition (20, 21) and hence their thixotropic elastoviscoplastic (TEVP) properties. The term *thixotropic* means that the fluid shows a time-dependent response even under a fixed level of force, as a finite amount of time is required for the highly filled particulate phase in the fluid to form a microstructure or for the microstructure to be destroyed. On the other hand, the term *elastoviscoplastic* means that the fluid shows rheological responses between a viscous fluid and an elastoplastic solid (40–45). As the dispensing nozzle retracts, the liquid bridge is extended, stretching polymer molecules in the fluid. The induced elastic restoring force tends to resist extensional deformation, making the liquid bridge more stable and harder to break (22, 23). Furthermore, the high-volume fraction of particulates dispersed in the fluid can form a microstructure able to store energy, causing the fluid to behave like an elastic solid under low stress. A minimum amount of stress, namely the “yield stress,” is required to generate flow in the fluid. Consequently, the dispensing nozzle must be retracted over a minimum distance so that the liquid bridge becomes thin enough and the capillary force strong enough to overcome the yield stress (46, 47), further worsening stringing.

### Edge fracture

Various strategies have been employed to prevent stringing and improve dispensing quality. The simplest is to introduce a dwell time after dispensing to allow the sagging of fluid before the nozzle retracts and moves to its new position (20, 21). However, this method renders the dispensing process much slower and does not apply to fluids with high yield stress. A more labor-intensive method is to alter the rheological properties of the fluid (1, 3). However, this limits the kinds of fluid that can be dispensed. A compromise between the dispensing speed, precision, and the types of fluids dispensable seems unavoidable. Is it even possible to dispense a rheologically complex fluid quickly and cleanly without sacrificing its rheological properties? Our answer is affirmative—with edge fracture.

Edge fracture is a flow instability often observed in rotational rheometry when an elastic fluid is subjected to a sufficiently strong shear (48–59). The phenomenon is characterized by the sudden indentation of the fluid’s free surface, which can invade the fluid sample and render rheological measurement results at high shear rates invalid. Because of this, rheologists have come up with different ways, such as liquid metal sealing (60, 61), specially designed guard rings (62), and the cone-partitioned plate geometry (63–65), to minimize the negative effects of edge fracture on their experiments. Recently, constructive efforts have been made to harness the phenomenon to destabilize liquid bridges. In previous works with viscoelastic fluids (66, 67), we showed that the indentation induced by edge fracture could propagate towards the liquid bridge center and cause its radius to undergo power-law decay. This quickly creates a clean cut in the liquid bridge’s midplane, eliminating the need to retract the dispensing nozzle and extend the bridge. Here, to show that edge fracture can be used to solve the stringing problem, we study the torsional deformation of liquid bridges made of a TEVP paste representative of those particulate composites used in the electronic packaging industry (20, 21).

### Our approach

**Fig. 1.**
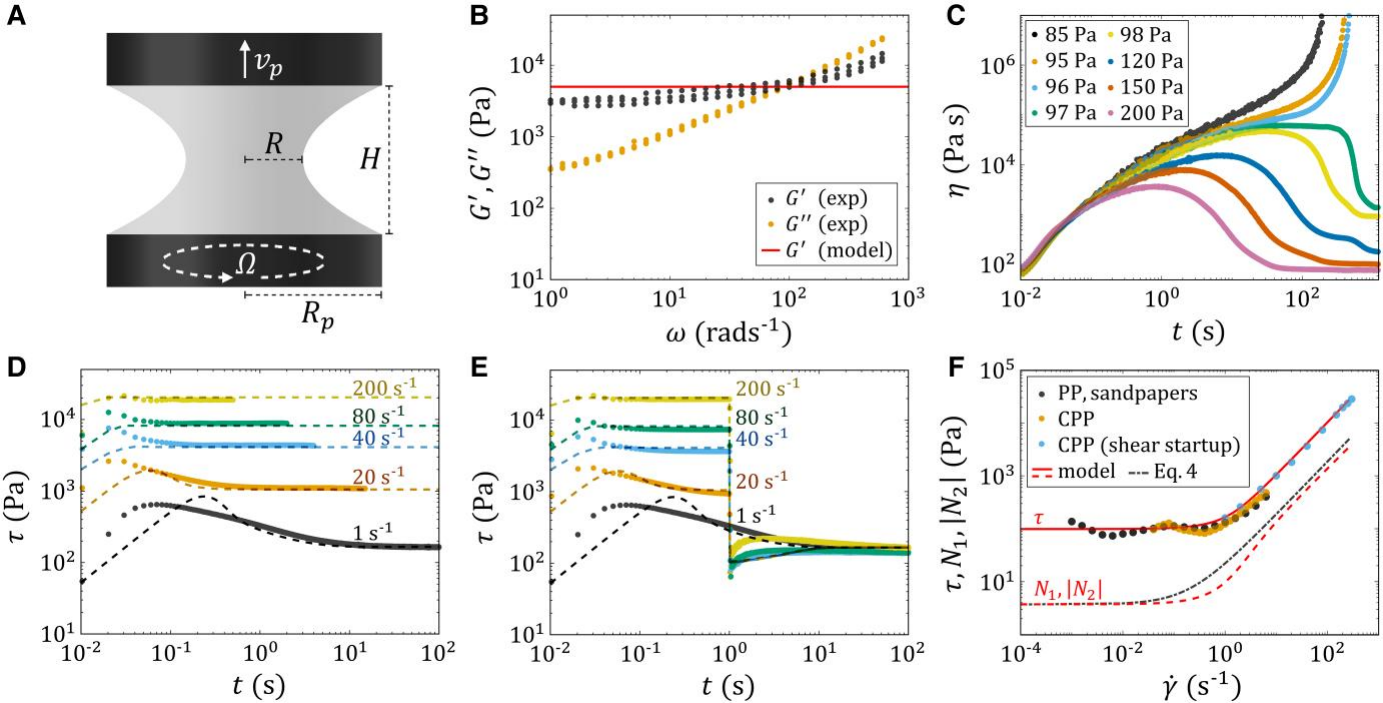
A) Schematic of the liquid bridge configuration. The top plate can move axially at a speed of $v_{p}$; the bottom plate can rotate unidirectionally at a rotational speed of Ω. B) SAOS test results of the thermal paste, showing the storage modulus $G^{\prime }$ and loss modulus $G^{\prime \prime }$ as functions of angular frequency ω. C) Creep test results of the thermal paste, showing the shear viscosity η as a function of time t under different applied step shear stress σ. D) Shear startup test results of the thermal paste, showing the shear stress τ as a function of time t under different applied step shear rates $\dot{\gamma }$. E) Two-step shear test results of the thermal paste, showing the shear stress τ as a function of time t under different applied shear rates $\dot{\gamma }(t)$. A step shear rate of different magnitudes (legend) is first applied to the paste for 1 s, followed by another step shear rate of 1 s${}^{-1}$ for 300 s. F) Steady shear test results of the thermal paste, showing the steady-state shear stress τ and normal stress differences $N_{1}$, $\left|N_{2}\right|$ as a function of applied shear rate $\dot{\gamma }$. PP and CPP are abbreviations of the parallel plate and the cone-partitioned plate geometries, respectively. For B–F), solid symbols are experimental results, while solid and dashed lines are data fits obtained by nonlinear regression of the TEVP model described in section sec. For F), the dash-dotted line represents the approximate normal stress response described by equation 4.

We consider a setup consisting of a TEVP liquid bridge sandwiched between two coaxial circular parallel plates. The top plate can move vertically, modeling the dispensing nozzle. The bottom plate can rotate unidirectionally to apply torsion to the liquid bridge (Fig. 1A).

The rheology of the TEVP fluid is characterized using the oscillatory shear test, creep test, shear startup test, two-step shear test, and steady shear test (Fig. 1B–F). The results can be mathematically described by a nonlinear, phenomenological TEVP constitutive model (lines in Fig. 1B–F). The set of equations relating the stress to the deformation rate tensor is given as


(1) $$(\frac{△\tau }{G_{t}})+\frac{\tau }{\eta_{t}}=\dot{\gamma }$$

(2) $$\frac{Ds}{Dt}=k_{1}(1-s)-k_{2}\dot{\gamma }\;:\frac{\tau }{G_{t}}s.$$

Here, τ is the deviatoric stress tensor and $\dot{\gamma }=\nabla u+(\nabla u{)}^{T}$ is the deformation rate tensor, where u is the velocity vector. The triangle above $\tau /G_{t}$ in Eq. 1 is the lower convected derivative. The material derivative is D/Dt=∂/∂t+u⋅∇. Additionally, s is a nondimensional structural parameter, representing a simplified description of the material’s instantaneous degree of structure. When s=1, the material resembles an elastic solid. When s→0, the material resembles a weakly viscoelastic fluid. Detailed descriptions regarding the role of s and its application to structural kinetics models of thixotropy are given by Larson and Wei (68).

The constitutive model has six material parameters: the thixotropic viscosity $\eta_{t}=\eta_{\;pl}/(1-s)$, which depends on the plastic viscosity $\eta_{\;pl}$ and the structural parameter s, the thixotropic elastic modulus $G_{t}=G(1+m/s^{n})$, which depends on the elastic modulus G, s, and two adjustable parameters m and n. The parameters $k_{1}$ and $k_{2}$ are related to the rate of the structural build-up and breakage of the material’s microstructure. As can be seen in Eq. 1, when s=1, the term $\tau /\eta_{t}\equiv (1-s)\tau /\eta_{\;pl}$ vanishes and the TEVP model reduces to the neo-Hookean elastic model. On the other hand, as s→0, the material structure is destroyed, corresponding to a viscosity plateau $\eta_{t}\to \eta_{\;pl}$ and a vanishing elasticity ${G_{t}}^{-1}\to 0$. The latter argument is supported by the fact that the elasticity in elastoviscoplastic fluids stems from the self-assembly of the microstructure into a stress-bearing network (69). When this network collapses, the physical interactions within the microstructure weaken, diminishing elasticity entirely (70). When 0<s<1, the material behaves like a thixotropic viscoelastic fluid, whose viscosity and elastic modulus depend on the deformation history under the isotropic hardening mechanism.

Although the model does not include an explicit yield stress parameter, a stress plateau can be observed in simple shear as $\dot{\gamma }\to 0$ (Fig. 1F). This stress plateau originates from the definition of the thixotropic viscosity and defines the plastic deformation regime. The value of the stress plateau in simple shear, or the yield stress, can be expressed as


(3) $$\tau_{y}=\sqrt{\frac{G(1+m)\eta_{\;pl}k_{1}}{2k_{2}}}.$$

Meanwhile, the normal stress differences as a function of the shear rate $\dot{\gamma }$ can be approximated as


(4) $$N_{1}=-N_{2}=\eta_{\;pl}\frac{k_{1}}{k_{2}}+\eta_{\;pl}\sqrt{\frac{2\eta_{\;pl}k_{1}}{Gmk_{2}}}\dot{\gamma }.$$

As $\dot{\gamma }\to 0$, $N_{1}=-N_{2}$ approach the plateau value $\eta_{\;pl}k_{1}/k_{2}$. As $\dot{\gamma }\to \infty$, $N_{1}=-N_{2}$ scale linearly with the shear rate $\dot{\gamma }$ (see solid black line in Fig. 1F), which is a common rheological feature of non-Brownian particulate suspensions (71).

The constitutive model, coupled with the mass and momentum conservation equations and suitable initial and boundary conditions, allows the TEVP liquid bridge to be numerically simulated, complementing the analysis of the experimental results. Details of the experimental protocol, material characterization, constitutive modeling, and simulation setup can be found in section “Materials and methods.”

First, we show that liquid bridges made of the TEVP fluid exhibit extensional behavior unfavorable to the nozzle retraction method of inducing breakup. For a typical liquid bridge of radius O (1 mm), lifting the top plate at a speed of O (1 mm s${}^{-1}$) will cause the liquid bridge to break up in O (10 s). However, a long capillary tail will also form, leading to the stringing problem, which worsens as the top plate is lifted faster. Then, we show that applying torsion to the TEVP liquid bridge can induce edge fracture, creating an indent on the liquid bridge’s free surface propagating radially inwards. By edge fracture, the breakup time of the TEVP liquid bridge decreases in a power-law-like fashion with respect to the rotational speed of the bottom plate. For a liquid bridge of radius O (1 mm), rotating the bottom plate at a speed of O (100 rad s${}^{-1}$) will cause the liquid bridge to break up in O (1 s). More importantly, edge fracture can destabilize TEVP liquid bridges with an initial height-to-neck radius ratio as small as 0.7, showing its great potential to be employed in developing new nozzles to dispense rheologically complex fluids quickly and cleanly, avoiding the stringing problem.

## Results

### TEVP liquid bridge under extension

**Fig. 2.**
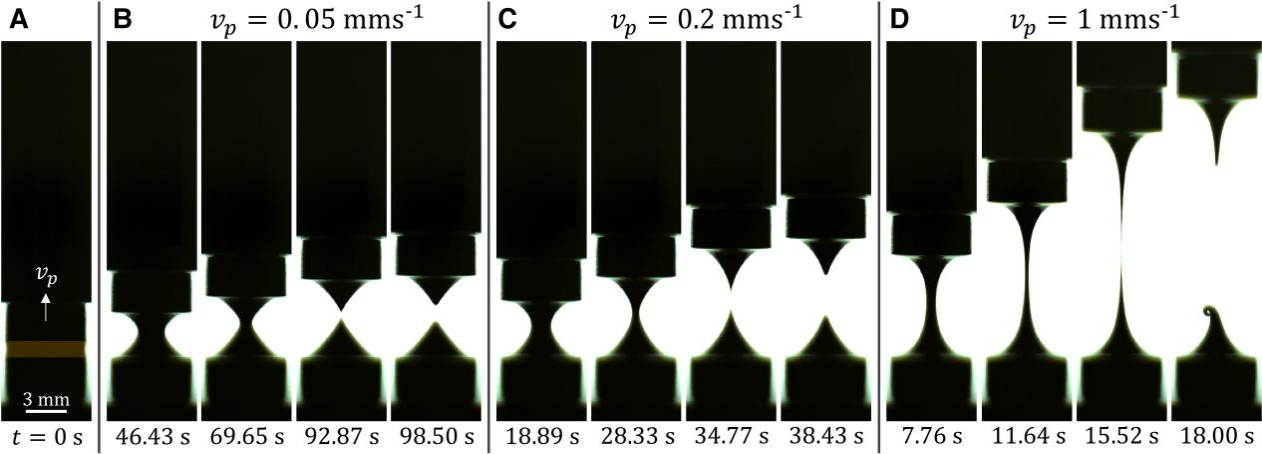
Snapshots of the TEVP liquid bridge subjected to different speeds $v_{p}$ of the top plate captured at different times t. A) t=0 s. The initial height of the liquid bridge is $H_{i}=1$ mm. B) $v_{p}=0.05$ mm s${}^{-1}$. C) $v_{p}=0.2$ mm s${}^{-1}$. D) $v_{p}=1$ mm s${}^{-1}$.

The extensional behavior of the TEVP liquid bridge depends upon the speed $v_{p}$ at which the top plate is lifted, as shown in Fig. 2. The liquid bridge configuration at t=0 s is shown in Fig. 2A. For $v_{p}=0.05$ mm s${}^{-1}$ (Fig. 2B), initially at t=46.43 s as the top plate retracts, the liquid bridge is of a rather parabolic shape. As time progresses to t=69.65 s, a more conical shape can be seen. At t=92.87 s, the liquid bridge breaks up, separating into two liquid reservoirs, each with a sharp conical tip on the top and bottom plates. A moment later, at t=98.5 s, the liquid reservoirs retain their conical shapes, except that their previously sharp tips become blunt because of surface tension.

For $v_{p}=0.2$ mm s${}^{-1}$ (Fig. 2C), the deformation of the liquid bridge is similar to that of $v_{p}$ to 0.05 mm s${}^{-1}$, except that the liquid bridge is seen to break up at a larger height and in a shorter time of 34.77 s. Also, the conical tips of the liquid reservoirs immediately after the breakup are sharper and more tapered. Increasing $v_{p}$ to 1 mm s${}^{-1}$ (Fig. 2D) further amplifies these differences. For instance, at t=11.64 s, the liquid bridge can be extended to a greater length without breakup compared to the two previous cases (see Fig. 2B and C). At t=15.52 s, when the liquid bridge pinches off, each liquid reservoir is seen to have a much sharper tip resembling a long tail. The long tail of the lower reservoir then collapses under its weight. The lower reservoir is seen to have a conical shape but with its apex bending over, manifesting the stringing problem when fluid is dispensed by the nozzle retraction method.

**Fig. 3.**
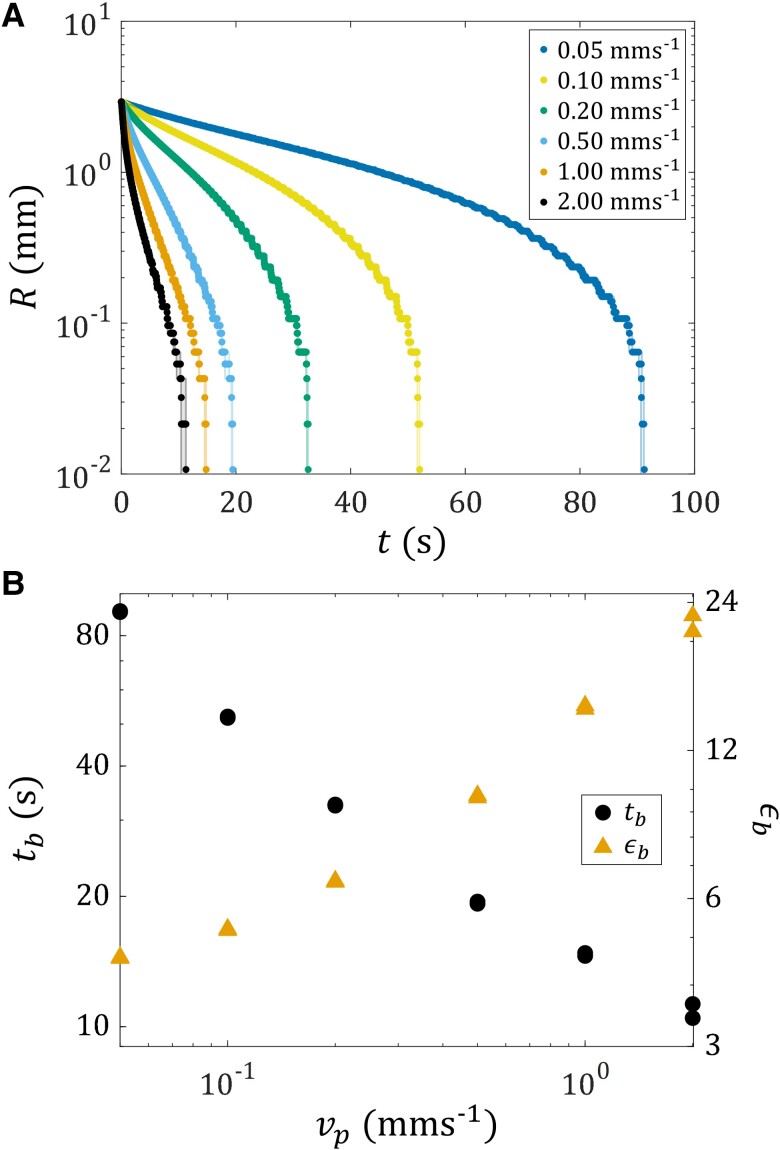
A) Experimentally measured neck radius R as a function of time t of the TEVP liquid bridge subjected to different speeds $v_{p}$ of the top plate. Solid symbols are data averaged between two experimental trials. Half-transparent solid lines are data obtained from each of the two experimental trials. B) Experimentally measured breakup time $t_{b}$ and extensional strain-to-break $\epsilon_{b}=v_{p}t_{b}/H_{i}$, where $H_{i}=1$ mm, of the liquid bridge as a function of $v_{p}$. For each $v_{p}$, data from two experimental trials are shown, which overlap with each other.

To inspect the extensional behavior quantitatively, Fig. 3A shows the neck radius R of the liquid bridge as a function of time t for different speeds $v_{p}$ of the top plate. As a general trend, R tends to decay faster as $v_{p}$ increases, which can be explained by volume conservation. However, this is no longer true as R decreases below around 0.1 mm, at which point R drops abruptly towards zero in all cases. Fig. 3B further shows the breakup time $t_{b}$ and the extensional strain-to-break $\epsilon_{b}=v_{p}t_{b}/H_{i}$ as a function of $v_{p}$. Consistent with what was observed in Fig. 2, $t_{b}$ decreases and $\epsilon_{b}$ increases as $v_{p}$ increases. Moschopoulos et al. (72) consider this behavior in their simulation of viscoplastic filament stretching. This behavior can be attributed to the greater viscous force exerted on the liquid bridge when $v_{p}$ is higher, causing more yielding in the liquid bridge. This enables more fluid to be brought upward and leads to a long bridge as the top plate retracts, making the stringing problem more severe.

### TEVP liquid bridge under torsion: effects of Ω

**Fig. 4.**
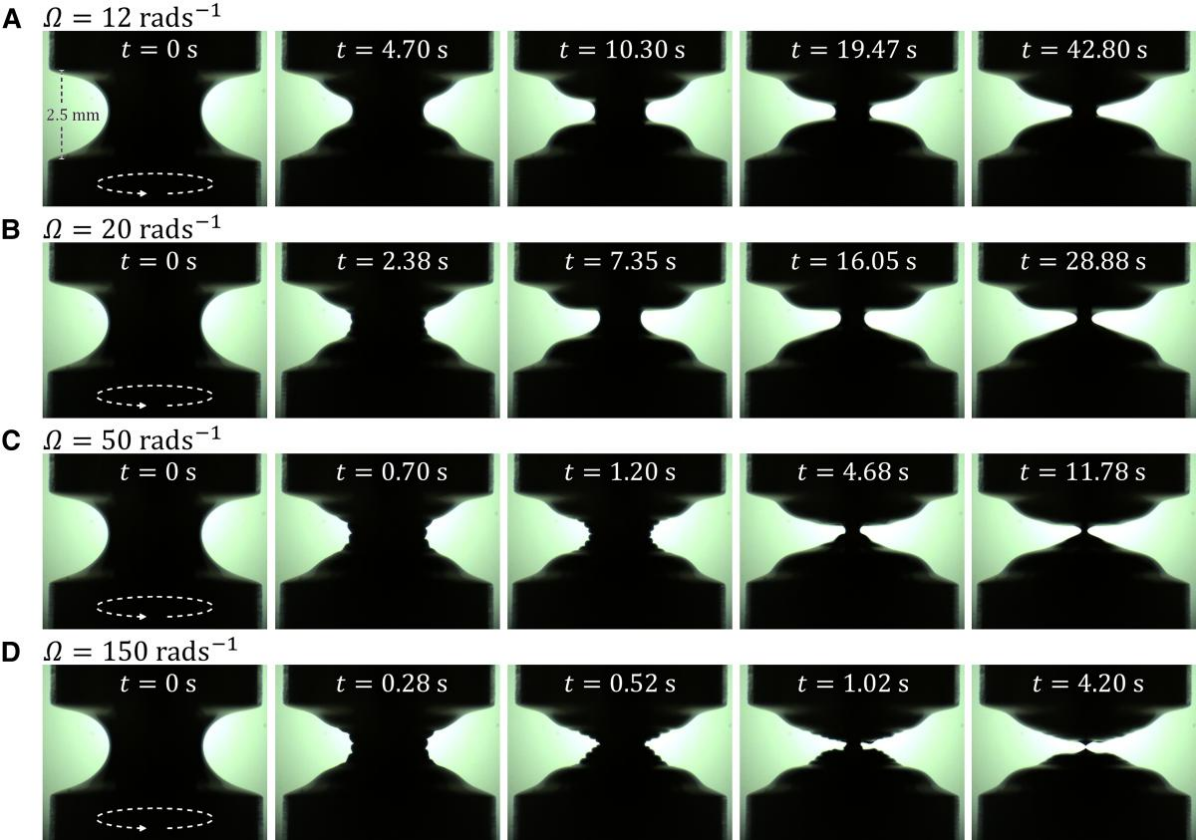
Snapshots of the TEVP liquid bridge subjected to different rotational speeds Ω of the bottom plate captured at different times t. The bottom plate rotates counter-clockwise when viewed from above. The height of the liquid bridge is H=2.5 mm. A) Ω=12 rad s${}^{-1}$. B) Ω=20 rad s${}^{-1}$. C) Ω=50 rad s${}^{-1}$. D) Ω=150 rad s${}^{-1}$.

The stringing problem can be prevented by using torsion instead of an extension to destabilize the liquid bridge. Fig. 4 shows snapshots of the TEVP liquid bridge of height H=2.5 mm subjected to different rotational speeds Ω of the bottom plate at different times t. The liquid bridge is stable with such a low height, meaning it does not break up under surface tension and gravity alone. For Ω=12 rad s${}^{-1}$ (Fig. 4A), at t=0 s when the rotation is just started, the liquid bridge adopts an approximately parabolic shape. Later at t=4.7 s, a smooth and semicircular indent on the liquid bridge surface can be seen. The indent then propagates towards the liquid bridge center, during which its radius of curvature decreases. Finally, for t≥42.8 s, the indent propagation halts, and the liquid bridge seems to have reached a steady state. In the entire thinning process, the liquid bridge is symmetric around its vertical centerline and across its horizontal midplane.

For Ω=20 rad s${}^{-1}$ (Fig. 4B), at t=2.38 s an indent can also be seen. However, its surface appears wrinkled instead of smooth, suggesting that the liquid bridge has broken its rotational and horizontal symmetries. As time proceeds to t=7.35 s, the characteristic size of the wrinkles becomes so small that practically it cannot be discerned; the liquid bridge has regained its rotational symmetry. Nonetheless, its horizontal symmetry is still lost, as there is more fluid on the bottom than on the top plate. The indent then continues to propagate until the process halts at t=28.88 s. At this point, the neck radius of the liquid bridge is smaller than that in the previous case of Ω=12 rad s${}^{-1}$ (see t=42.80 s, Fig. 4A).

Increasing Ω to 50 rad s${}^{-1}$ (Fig. 4C), at t=0.70 s an even more wrinkled indent can be seen. On its surface, ∼3 wrinkles can be spotted. At t=1.2 s, the wrinkles appear to have decreased in size and increased in number to ∼7. Such an increase in the number of wrinkles is attributed to the rotation of the bottom plate. In the time interval of Δt=1.2−0.7=0.5 s, the bottom plate would complete ΩΔt/2π≈4 cycles of rotation, which correlates very well with the 7−3=4 extra minima observed at t=1.2 s. As time proceeds to t=4.68 s, the wrinkles have disappeared. The rotational symmetry of the liquid bridge is regained, but the horizontal symmetry is still lost. The indent propagation then halts at t=11.78 s, at which time the liquid bridge radius is smaller than the two previous cases of lower Ω (see t=42.8 s, Fig. 4A and t=28.88 s, Fig. 4B).

Further increasing Ω to 150 rad s${}^{-1}$ (Fig. 4D), at t=0.28 s, the shape of the wrinkled indent closely resembles the one seen in the Ω=50 rad s${}^{-1}$ case (see t=0.7 s, Fig. 4C). Then, at t=0.52 s, the indent has roughly the same number of wrinkles as that in Fig. 4C at t=1.2 s. However, the indent has become sharper instead of staying semicircular as a whole. Later at t=1.02 s, the liquid bridge radius is comparable to that seen in Fig. 4C at t=4.68 s. Nonetheless, wrinkles can still be seen on the indent’s surface at this higher rotational speed. Finally, at t=4.20 s, the liquid bridge pinches off, creating a rather smooth liquid reservoir on each plate. As a side note, if the rotation is stopped during the indent propagation process, the indent will stop propagating, rebound backward, and stay stationary. Such behavior is caused by the surface tension and yield stress of the TEVP fluid, as surface tension is the only force that can drive the indent backward, and yield stress is the only force that can counteract the effect of surface tension after rotation is stopped.

**Fig. 5.**
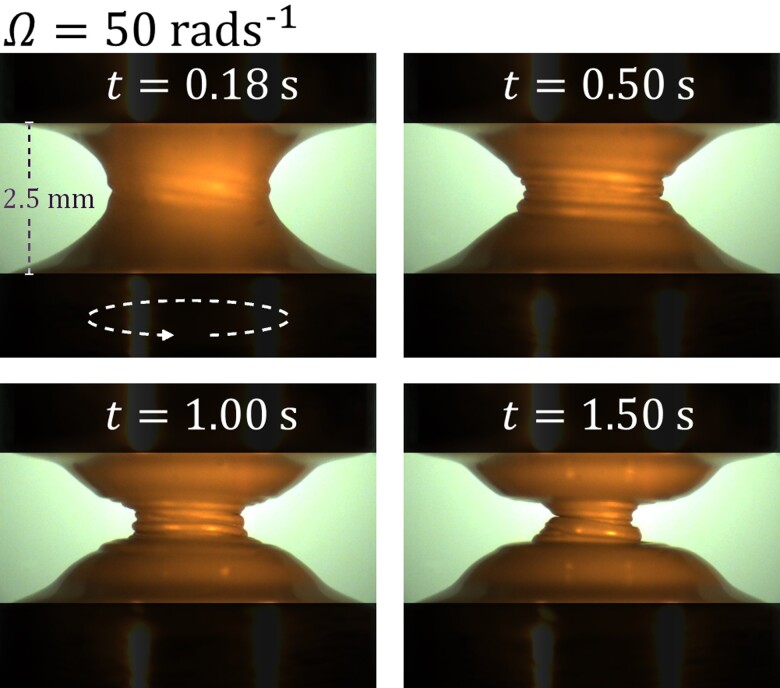
Snapshots of the TEVP liquid bridge subjected to a Ω=50 rad s${}^{-1}$ of the bottom plate captured at different times t, with the front surface of the liquid bridge illuminated. The bottom plate rotates counter-clockwise when viewed from above. The height of the liquid bridge is H=2.5 mm.

As mentioned above for Fig. 4B–D, the TEVP liquid bridge tends to have its horizontal symmetry broken as the wrinkled indent propagates. However, such behavior is, in fact, somewhat random. As the experiment is repeated, the horizontal symmetry can often be regained as the wrinkles disappear. Occasionally, more fluid can be seen on the top plate instead of the bottom one. To explain how the wrinkling may be related to the horizontal symmetry breaking behavior, Fig. 5 shows additional snapshots of the liquid bridge for the Ω=50 rad s${}^{-1}$ case with its front surface illuminated. At t=0.18 s, a slight disturbance can be spotted on the somewhat parabolic liquid bridge surface. As the bottom plate rotates, the disturbance twists around the liquid bridge. Because of this, at t=0.5 s and t=1 s, helical wrinkles can be seen on the liquid bridge surface. As the rotation continues, the number of turns of wrinkles increases. Also, as the indent keeps propagating, the wrinkles are forced to be in an ever-smaller space. These two effects together cause the wrinkles to shrink in size over time. This can amplify the Laplace pressure acting on the wrinkles, increasing their tendency to merge and hence the liquid bridge’s chance to break its horizontal symmetry, as observed in Fig. 5 at t=1.5 s. To show the entire liquid bridge thinning process, videos corresponding to the results of Ω=20, 50, and 150 rad s${}^{-1}$ with the liquid bridge’s front surface illuminated are shown in Movies S1–S3 in the SI Appendix, respectively.

**Fig. 6.**
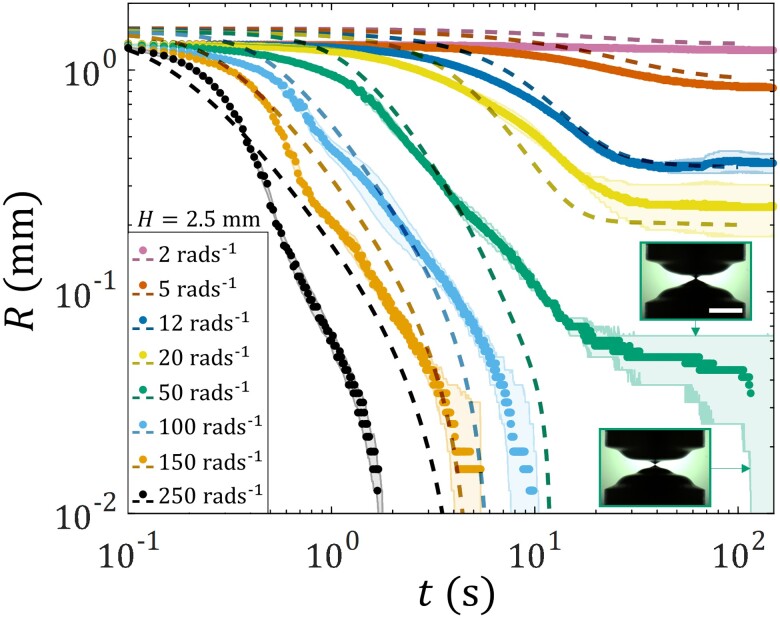
Experimentally measured and simulated neck radius R as a function of time t of the TEVP liquid bridge subjected to different rotational speeds Ω of the bottom plate. Solid symbols are data averaged between two experimental trials. Half-transparent solid lines are data obtained from each of the two experimental trials. Dashed lines are data obtained from the axisymmetric TEVP flow simulation. Inset images show the two possible fates of the liquid bridge for Ω=50 rad s${}^{-1}$. The upper insert image shows the case where the liquid bridge reaches a steady state. Scale bar: 2 mm. The lower insert image shows the case where the liquid bridge breaks up.

Having described qualitatively how the liquid bridge deforms under different levels of torsion, we next discuss the quantitative features. Fig. 6 shows the neck radius R(t) of the liquid bridge of H=2.5 mm subjected to different Ω. For Ω=2 rad s${}^{-1}$, torsion has virtually no effect on the liquid bridge radius, and R remains constant during the whole experimental period. As Ω is increased above 5 rad s${}^{-1}$, R can be seen to undergo power-law-like decay at a rate that increases with increasing Ω.

For Ω≥12 rad s${}^{-1}$, because of the wrinkled indent (see Fig. 5, for example), the measurement of the liquid bridge radius has an inherent variability and uncertainty. Such uncertainty is evident, especially near the end of the power-law-like decay process, where fluctuations (half-transparent solid lines) can be seen around the mean values of R (solid symbols). For Ω≤20 rad s${}^{-1}$, the power-law-like decay halts, and R plateaus when t reaches O (10 s), signifying that the liquid bridge has reached a steady state. For a larger Ω, the plateau level of R is seen to be smaller. On the other hand, for Ω≥100 rad s${}^{-1}$, the power-law-like decay continues until the liquid bridge breaks up. The case of Ω=50 rad s${}^{-1}$ shows both behaviors. As the experiment is repeated, the liquid bridge can either reach a steady state or break up. Fig. 6 also contains R(t) obtained from the axisymmetric TEVP flow simulation (dashed lines). The simulated curves agree reasonably well except for Ω=50 rad s${}^{-1}$ at which there is variability in the experimental behavior (see Fig. 4). Such an agreement provides us confidence regarding the simulated stress fields and structure in the liquid bridge, which will be discussed later in the main text.

### TEVP liquid bridge under torsion: effects of H

**Fig. 7.**
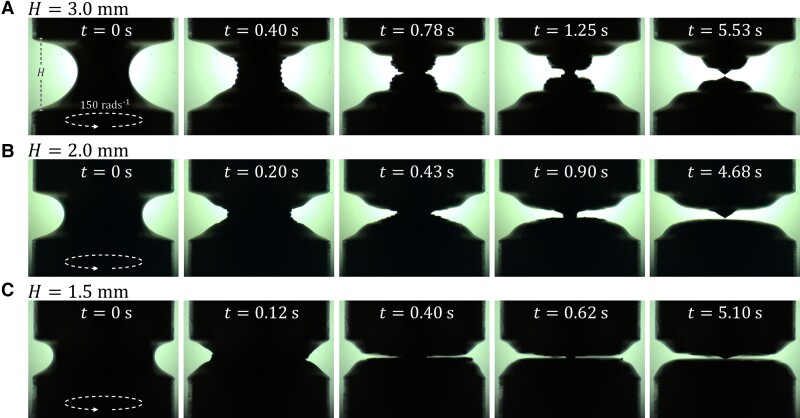
Snapshots of the TEVP liquid bridge of different heights H subjected to a rotational speed of Ω=150 rad s${}^{-1}$ of the bottom plate captured at different times t. The bottom plate rotates counter-clockwise when viewed from above. A) H=3 mm. B) H=2 mm. C) H=1.5 mm.

By decreasing the height H, it is possible to control more precisely the vertical position at which the liquid bridge pinches off. Fig. 7 shows snapshots illustrating the time evolution of TEVP liquid bridges of different H subjected to Ω=150 rad s${}^{-1}$. For H=3 mm (Fig. 7A), the liquid bridge is more slender than that for H=2.5 mm (see Fig. 4D). Because of this, more turns of the helical wrinkles can develop on the indent’s surface, rendering the breakup position of the liquid bridge more uncertain at later times. Here, only a specific case where the liquid bridge breaks up in the middle is shown. As the experiment is repeated, the situation can differ depending on how the wrinkles merge during the liquid bridge thinning process.

Decreasing H to 2 mm (Fig. 7B), fewer wrinkles can be seen on the indent’s surface. Hence, the liquid bridge’s breakup position is less uncertain than for higher H. For instance, at t=0.2, 0.43, and 0.9 s, the indent locates in the middle of the liquid bridge. However, at t=4.68 s, the upper liquid reservoir retains a small fluid tip after the liquid bridge breaks up. Such a fluid tip appears randomly on the upper or the lower reservoir as the experiment is repeated.

Further decreasing H to 1.5 mm (Fig. 7C), the space where the wrinkles can develop is further confined. As a result, the indent always sets in the same position and propagates along a similar path. Also, it is seen to be much sharper. At t=0.4, 0.62, and 5.1 s, the upper and lower surfaces of the indent are almost parallel to each other, creating a clean horizontal cut in the liquid bridge midplane as it breaks up. A small fluid tip is attached to the upper liquid reservoir; however, its size is smaller than in the H=2 mm case.

**Fig. 8.**
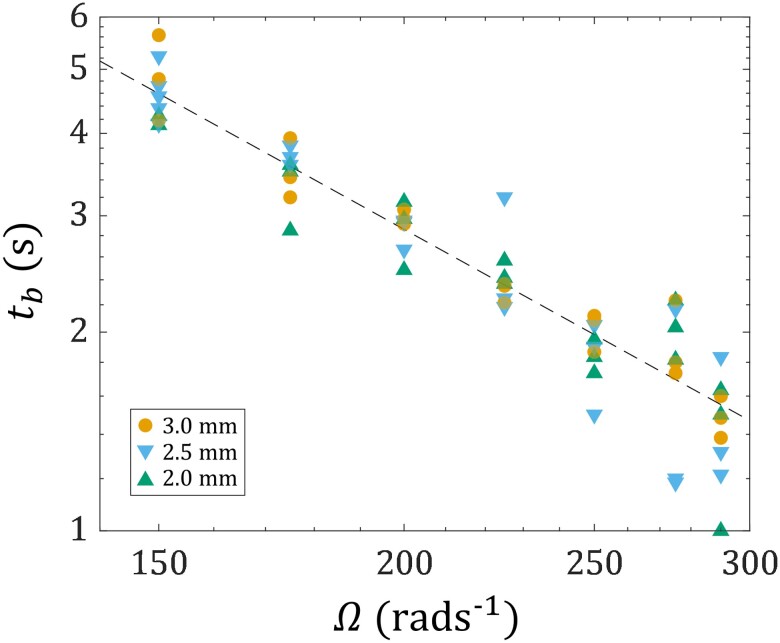
Experimentally measured breakup time $t_{b}$ of the TEVP liquid bridge of different heights H as a function of the bottom plate’s rotational speed Ω. For each Ω, raw data from at least three experimental trials are shown. The dashed line $t_{b}=17457\Omega^{-1.65}$, obtained by fitting a power-law to all the data points, is the visual guide showing how $t_{b}$ varies with Ω irrespective of H.

Fig. 8 further shows the breakup time $t_{b}$ of the liquid bridge with different H as a function of Ω. The data for H=1.5 mm are not shown, as the indent is so thin that precisely resolving $t_{b}$ is difficult by our current experimental setup, which can only capture the silhouette of the liquid bridge. As a general trend, $t_{b}$ decreases as Ω increases. Also, $t_{b}$ is insensitive to H, as the data points overlap. Hence, while the qualitative effect of H on the liquid bridge pinch-off process is significant (see Fig. 7), its quantitative effect is not. It is worthwhile to mention that the values of $t_{b}$ observed here are comparable to, or shorter than, those in the extensional case (see Fig. 3B), even though no extension is applied to the liquid bridge. This showcases the potential of using torsion to replace or complement extension in destabilizing TEVP liquid bridges quickly and cleanly, hence overcoming the stringing problem.

### Dimensional analysis

The liquid bridge deformation shown in Figs. 7–7 is an inherently complex process. It depends on the interaction between the inertial, elastic, plastic, capillary, viscous, gravitational, and thixotropic effects, which are related to the liquid bridge radius R, the rotational speed Ω, the characteristic length-scale a over which shear is distributed in the liquid bridge, the fluid density ρ=2500 kg m${}^{-3}$, the elastic modulus G(1+m)=5225 Pa, the plastic viscosity $\eta_{\;pl}=102$ Pa s, the yield stress $\tau_{y}=99$ Pa, the surface tension σ=20.6 mN m${}^{-1}$, and the gravitational acceleration g=9.81 m s${}^{-2}$. Details regarding how the materials parameters are obtained can be found in section “Materials and methods.”

For a liquid bridge subjected to torsion, the characteristic stress is $\tau_{c}=\eta_{\;pl}R\Omega /a$. Five dimensionless numbers can be obtained by comparing $\tau_{c}$ to the potentially relevant stresses of the TEVP liquid bridge deformation process; their orders of magnitude will inform us how relevant the stresses are. The Reynolds number $\text{Re}=\rho R^{2}\Omega^{2}/\tau_{c}$ characterizes the relative importance of the inertial and viscous effects. The Weissenberg number $\text{Wi}=\tau_{c}/G(1+m)$ characterizes the relative importance of the elastic and viscous effects. The Bingham number $\text{Bn}=\tau_{y}/\tau_{c}$ characterizes the relative importance of the plastic and viscous effects. The capillary number $\text{Ca}=\tau_{c}a/\sigma =\eta_{\;pl}R\Omega /\sigma$ characterizes the relative importance of the viscous and capillary effects. The Galilei number $\text{Ga}=\rho ga/\tau_{c}$ characterizes the relative importance of the gravitational and viscous effects.

Regarding the thixotropic effect, recall from Eq. 2 that the build-up and breakage rates of the microstructure in the TEVP fluid scale with $k_{1}$ and $[k_{2}/G(1+m)](R\Omega /a)\tau_{c}=[k_{2}/G(1+m)\eta_{\;pl}]\tau_{c}^{2}$, where $k_{1}=0.1$ s${}^{-1}$ and $k_{2}=2.74$. The ratio of the two rates allows the Mnemosyne number (73), which measures the relative speed of the structural build-up and breakage, to be defined as $\text{My}=[k_{2}/k_{1}G(1+m)\eta_{\;pl}]\tau_{c}^{2}$. However, since $\tau_{y}=\sqrt{G(1+m)\eta_{\;pl}k_{1}/2k_{2}}$ (Eq. 3), the Mnemosyne number can be rewritten as $\text{My}=1/2{\text{Bn}}^{2}$, which is essentially an inverse square Bingham number. This relation points out that plasticity is directly linked to the structural rearrangement inside the TEVP fluid. For Bn≫O(1) where the flow strength is weak, jamming (or attractive forces) in the material’s particulate phase dominates, leading to an elastic solid-like response. On the other hand, for Bn≪O(1) where the flow strength is strong, the material’s structure is destroyed, leading to the flow of the noninteracting particulate phase, corresponding to a weakly viscoelastic fluid-like response.

The problem seems somewhat complicated as five dimensionless numbers are involved. However, such complexity can be reduced by a simple order of magnitude analysis. To obtain an upper bound for Re, we assume that a=R and Ω=290 rad s${}^{-1}$, which is the highest rotational speed considered in the current study. For a typical R=1 mm, this gives $\text{Re}\in O(10^{-3})$. To obtain an upper bound for Ga, we assume that Ω=5 rad s${}^{-1}$, the lowest rotational speed at which indent formation is observed (see Fig. 6); this gives $\text{Ga}\in O(10^{-2})$. Since the upper bound magnitudes of both Re and Ga are much smaller than O(1), they can be safely neglected. Hence, the liquid bridge deformation process only depends on Wi, Bn, and Ca.

An important physical insight can be gained by further inspecting the orders of magnitude of Wi, Bn, and Ca. Assuming that $\tau_{c}$ has the same order of magnitude as G(1+m), we get Wi∈O(1) and $\text{Bn}\in O(10^{-2})$ regardless of the value of a, which implies that Bn, and hence the structural build-up effect, is negligible. Now assuming an indent with a lower bound size of a=0.1 mm (for example, Fig. 7C), from $\tau_{c}=\eta_{\;pl}R\Omega /a$ we get a characteristic velocity of RΩ≈5 mm s${}^{-1}$. This corresponds to Ca∈O(10), which is rather close to O(1) and therefore is not negligible. For a larger indent of size a=1 mm (for example, see Fig. 4A–C), we get RΩ≈50 mm s${}^{-1}$ and Ca∈O(100), which is much larger than O(1) and hence is negligible.

All in all, it is evident from the above analysis that the indentation phenomenon is a viscoelastic instability governed by Wi. Bn and Ca are involved only when the applied $\tau_{c}$ becomes considerably smaller than G(1+m) and when the indent formed is very sharp, respectively.

Equivalently, the liquid bridge thinning problem can be analyzed using the Tanner number Tn, which characterizes the relative importance of the torsion-induced normal stress and capillary effects. In our previous study of silicone oil bridges, the second normal stress difference $N_{2}$ was found to be responsible for the liquid bridge deformation under torsion (66). The silicone oil had a zero-shear second normal stress coefficient $\Psi_{2,0}=0.1$ Pa s${}^{2}$ and the same surface tension as the TEVP fluid. A typical R=1 mm, a=0.1 mm, and Ω=5 rad s${}^{-1}$ give $\text{Tn}=\Psi_{2,0}(R\Omega /a{)}^{2}a/\sigma \in O(1)$. For the TEVP fluid, however, caution has to be paid, as the constitutive model predicts that the normal stress differences scale linearly rather than quadratically with the shear rate (see Fig. 1F). For a characteristic shear stress of $\tau_{c}>G(1+m)$, the normal stress differences are proportional to the shear stress with a proportionality constant $\sqrt{2\eta_{\;pl}k_{1}/Gmk_{2}}$ (see Eq. 4). In such a case, the Tanner number assumes the form $\text{Tn}=\eta_{\;pl}R\Omega \sqrt{2\eta_{\;pl}k_{1}/Gmk_{2}}/\sigma$. A typical R=1 mm and Ω=5 rad s${}^{-1}$ give Tn∈O(1), which agrees with the silicone oil case, signifying the importance of the torsion-induced normal stress in the liquid bridge deformation process.

### Relation to edge fracture

**Fig. 9.**
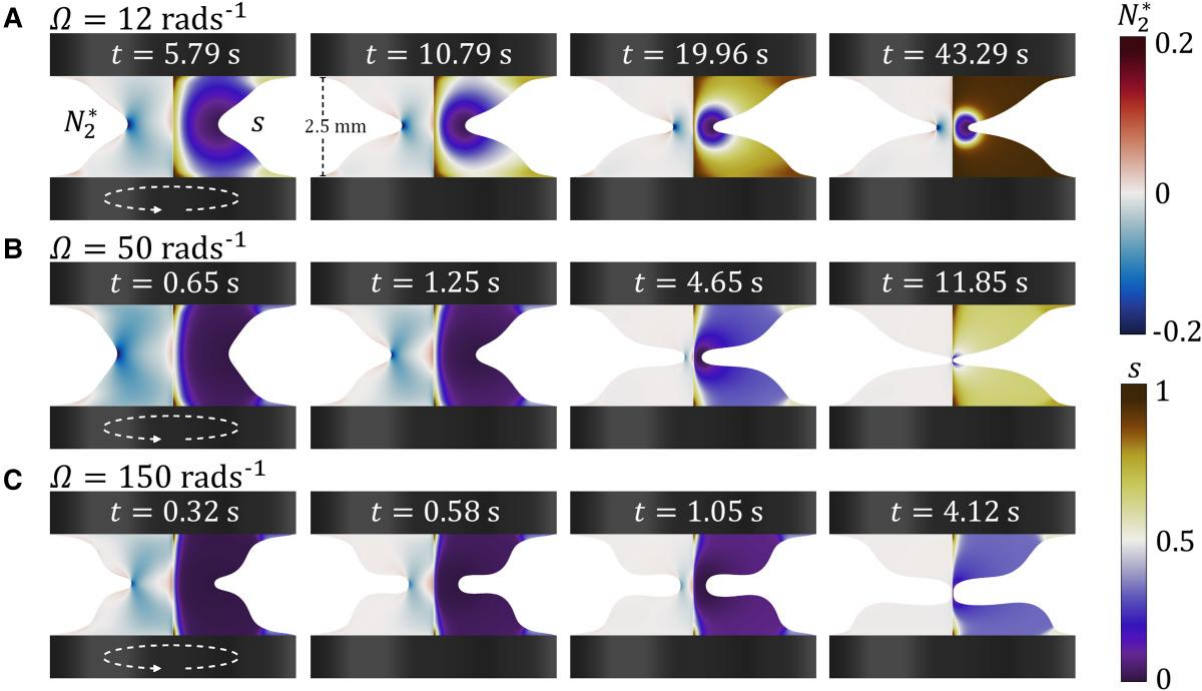
Simulated dimensionless second normal stress difference $N_{2}^{*}=N_{2}/\eta_{\;pl}\Omega$ (left) and structural parameter s (right) of the TEVP liquid bridge subjected to different rotational speeds Ω of the bottom plate at different times t. The height of the liquid bridge is H=2.5 mm. A) Ω=12 rad s${}^{-1}$. B) Ω=50 rad s${}^{-1}$. C) Ω=150 rad s${}^{-1}$.

The power-law-like decay behavior of the liquid bridge radius R (see Fig. 6) and the dependence on Tn lead to the supposition that the indentation phenomenon is caused by edge fracture (66). To inspect how the edge fracture of TEVP liquid bridges may be different from that occurring for viscoelastic fluids, Fig. 9 shows the simulated distributions of the dimensionless second normal stress difference $N_{2}^{*}=N_{2}/\eta_{\;pl}\Omega$ (left) and the structural parameter s (right) for bridges corresponding to those shown in Fig. 4. For all Ω, throughout the indent propagation process, a negative region of $N_{2}^{*}$ can be spotted at the indent’s tip. This agrees with the observation of Chan et al. (66), suggesting that the indent propagation process is indeed caused by edge fracture, as anticipated.

For Ω=12 rad s${}^{-1}$ (Fig. 9A), initially, at t=5.79 s, the structural parameter s is close to zero in almost the whole liquid bridge except around the vertical centerline and the rims of the parallel plates. This means that most microstructure in the liquid bridge is destroyed, and the bridge is in a fluid state. As time proceeds, the area where s≈0 shrinks towards the indent’s tip. Finally, at t=43.29 s, most of the fluid volume has s≈1 except at the indent’s tip, implying that the microstructure is rebuilt throughout most of the fluid. Such time evolution of the structural parameter s can be explained using the Bingham number Bn. In the initial stage of indent propagation, both R and a are of the same order of magnitude. For Ω=12 rad s${}^{-1}$, this gives $\text{Bn}\in O(10^{-2})$, meaning that the structural breakage effect is negligible and that the TEVP fluid behaves simply like a weakly viscoelastic fluid. In the later stage, as the indent has propagated, R∈O(0.1 mm) and a∈O(1 mm). This gives Bn∈O(0.1), which is rather close to O(1), signifying that plasticity and rebuild of the fluid’s microstructure start to affect the liquid bridge dynamics, increasing the area where s≈1.

For the higher Ω cases, it is important to remember that helical wrinkles are observed experimentally for Ω≥20 rad s${}^{-1}$ (see Fig. 4B–D). Therefore, the axisymmetry assumed in the simulation does not hold. In fact, the vertical symmetry breaking of the TEVP liquid bridge is not observed in the simulation for Ω≥20 rad s${}^{-1}$, supporting our previous assertion that the phenomenon is caused by the wrinkles formed on the indent. Despite the simulated results not corresponding precisely to the experiment, they may still complement the prediction of our dimensional analysis regarding how plasticity and thixotropy may affect the liquid bridge thinning process; hence it is worthwhile to describe them in some detail.

For Ω to 50 rad s${}^{-1}$ (Fig. 4B), the time evolution of s becomes different from the previous cases. An obvious example is at t=4.65 s. Although the liquid bridge has thinned significantly, s is still close to zero in most of the bridge volume. Such behavior is further amplified for Ω=150 rad s${}^{-1}$ (Fig. 4C). Even at the moment of pinch-off, s is close to zero in almost the entire bridge, meaning that the liquid bridge breakup has become faster than the structural rebuild process. This can again be rationalized by the Bingham number Bn. Assuming that R∈O(0.1 mm) and a∈O(1 mm) gives $\text{Bn}\in O(10^{-2})$, which is considerably smaller than O(1), meaning that structural rebuild has essentially no effects on the liquid bridge dynamics. This explains the experimental observation that the upper and lower liquid reservoirs’ surfaces tend to be smooth rather than wrinkled after the liquid bridge breakup (for example, see Figs. 4D and 7). As the liquid bridge is still fluid, surface tension can flatten the helical wrinkles on the bridge’s surface.

## Discussion and conclusion

In this work, we have considered a setup consisting of a TEVP liquid bridge sandwiched between two coaxial circular parallel plates. The top plate could be translated vertically at a speed of $v_{p}$; the bottom plate could be rotated unidirectionally at a rotational speed of Ω. Experiments revealed that the TEVP liquid bridge suffers from the stringing problem under extension. For an intermediate $v_{p}$, the liquid bridge broke into two conical liquid reservoirs on the top and bottom plates. Although the breakup time became shorter for a sufficiently high $v_{p}$, the minimum distance at which the liquid bridge broke up increased, forming a long capillary tail after pinch-off. In dispensing applications, such tails can fall randomly onto and contaminate the dispensing substrate or the printed structure.

Previous studies (66, 67) have demonstrated the effectiveness of using torsion to break viscoelastic liquid bridges cleanly and quickly. Building on this success, torsion was proposed as a solution to rapidly destabilize TEVP liquid bridges while addressing the stringing issue. We found that applying a sufficient level of torsion causes an indent to form on the liquid bridge’s free surface, which propagates towards the centerline of the bridge, resulting in a faster breakup than using extension. While helical wrinkles are observed in the later stages of the indent propagation process, their effects are currently unclear and merit further investigation. Dimensional analysis shows that the indent propagation depends on the interaction between the elastic, capillary, and plastic effects. When the elastic effect dominates, plasticity is negligible. The indent propagation phenomenon observed is hypothesized to be caused by edge fracture. Complementary numerical simulations confirm the proposed mechanism and show that as Ω increases, the liquid bridge becomes more fluid-like during the indentation process. This quantitative result may help better understand the edge fracture of suspensions (52, 53, 74–76), which has remained rather descriptive over the past few decades.

Although our focus is on the edge fracture of TEVP liquid bridges, the methodology employed can be readily used to understand other phenomena involving TEVP materials, which generally depend on the complex interaction between the inertial, elastic, plastic, capillary, viscous, gravitational, and thixotropic effects. For instance, the constitutive model presented in this study (Eqs. 1 and 2) provides a convenient framework to perform dimensional analysis. This enables us to identify the key stresses involved in the deformation process of the TEVP liquid bridges and to corroborate that the underlying mechanism is edge fracture. Perhaps more important is that, under the proposed framework, plasticity is directly linked to the structural rearrangements inside the TEVP material. By solving our model analytically, we found an expression that directly relates the yield stress, the elastic modulus, the plastic viscosity, and the ratio of the thixotropic build-up and breakage timescales. Using the formula (Eq. 3), one can directly obtain the ratio of the thixotropic timescales and define the Mnemosyne number My (73) simply by performing the steady shear and frequency sweep experiment. This can help experimentalists analyze their results more easily without fitting complex constitutive models to transient rheometric data.

In summary, our results provide new insights toward a better understanding of the free-surface flows of TEVP materials. They will inform new designs of nozzles to dispense complex fluids quickly and cleanly, which are important for various industrial and biotechnology applications. Nozzle designs with a rotatable printhead already exist (77); they can be modified to test the idea of using edge fracture to improve the dispensing quality of rheologically complex fluids.

## Methodology

### Experimental protocol

The experimental setup consists of a TEVP liquid bridge sandwiched between two coaxial circular parallel plates of radius $R_{p}=3$ mm (Fig. 1A). The plates are installed on a strain-controlled rotational rheometer (ARES-G2, TA instruments). The top plate can move vertically; the bottom plate can rotate unidirectionally. Due to the highly viscous nature of the TEVP fluid, positive displacement pipettes cannot be used to control the liquid bridge volume. Instead, the fluid is first loaded onto the bottom plate. Then, the gap between the top and bottom plates is decreased to $H_{0}=0.95$ mm, and the extra fluid is carefully trimmed.

To eliminate flow histories and ensure that the liquid bridge is axisymmetric, before each experiment, the liquid bridge is slightly stretched from $H_{0}$ to $H_{i}=1$ mm and presheared under an effective shear rate of $\dot{\gamma }=R_{p}\Omega /H_{i}=1$ s${}^{-1}$ for 180 s. It is then stretched from $H_{i}$ to a new height H at speed $v_{p}=0.1$ mm s${}^{-1}$, and allowed to relax for 600 s. At t=0 s, the bottom plate is accelerated to a rotational speed of Ω within 0.15 s. For the extensional experiments, the prestretching procedure is skipped. Following the preshear, the gap is maintained at $H_{i}$, and the fluid is let relaxed for 600 s. Then, at t=0 s, the liquid bridge is stretched continuously from $H_{i}$ at different speeds $v_{p}$ until it breaks. The reason for using $H_{i}$ instead of $H_{0}$ during the preshear is to ensure that the liquid bridge surface is concave. In this way, the normal stress induced in the preshear will be maximized at the neck of the liquid bridge, deforming the bridge surface in a reproducible manner and ensuring that the bridge will have the same initial surface profile in all experiments. Videos of the liquid bridge deformation process are captured and analyzed using the protocol described in Ref. (67). All experiments are performed at room temperature at 24 ${}^{\circ }$C.

### Material characterization

The TEVP fluid used is a thermal paste of >50 wt% alumina oxide and 5–10 wt% monocrystalline diamond dispersed in 40–45 wt% methyl silicone (MX-4 thermal compound, Arctic Inc.). Its non-evaporative and chemically stable natures allow experiments to be performed for an extended period, which is essential for the preshearing and relaxation procedures of the liquid bridge. The density of the paste is ρ=2500 kg m${}^{-3}$. The surface tension is assumed to be the same as that of methyl silicone, i.e. σ=20.6 mN m${}^{-1}$ (66). Six rheological measurement protocols are employed to characterize the TEVP fluid: oscillatory shear test, creep test, shear startup test, two-step shear test, and steady shear test. A strain-controlled rheometer (ARES-G2, TA instruments) is used for all rheological measurements performed at 24 ${}^{\circ }$C. To eliminate flow histories, before each measurement, the fluid sample is presheared under a shear rate of $\dot{\gamma }=1$ s${}^{-1}$ for 180 s and subsequently allowed to relax for 600 s.

A 40 mm diameter stainless steel parallel plate geometry is used for the oscillatory shear test and creep test. C800-grit sandpapers are fixed with double-faced tape to each plate to prevent sample wall slip. Both amplitude and frequency sweeps are performed for the oscillatory shear test. The amplitude sweep is performed with an oscillation frequency of ω=1 rad s${}^{-1}$ in a strain amplitude range of 0.01%≤γ≤100%, from which the linear viscoelastic regime is identified to be γ≤0.4%. The frequency sweep, *aka* the small amplitude oscillatory shear (SAOS) test, is performed with a strain amplitude of γ=0.1% in a frequency range of $0.1\;{\mathrm{rad}\;s}^{-1}\leq \omega \leq 600\;{\mathrm{rad}\;s}^{-1}$, from which the storage modulus $G^{\prime }$ and the loss modulus $G^{\prime \prime }$ are obtained (Fig. 1B). For ω<100 rad s${}^{-1}$, $G^{\prime }$ is seen to be larger than $G^{\prime \prime }$, signifying that the sample behaves like a viscoelastic solid at low frequencies and strain amplitudes, as expected for TEVP materials. For the creep test, a step shear stress τ of various magnitude is applied to the fluid sample for 1200 s, and the shear viscosity response η is recorded as a function of time t (Fig. 1C). If τ is larger than the yield stress, η will plateau as time proceeds; if it is smaller, η will tend to infinity instead (78). Such a bifurcation phenomenon of η allows us to determine the yield stress $\tau_{y}^{e}=97$ Pa unambiguously, taking into account the effect of thixotropy.

A 25 mm diameter stainless steel 0.1 rad cone-partitioned plate geometry is used for the shear startup and two-step shear tests. The geometry is a modification of the conventional cone and plate geometry in which only the central 10 mm diameter of the plate is coupled to the stress transducer. The remaining 15 mm diameter of the fluid sample acts as a guard-ring to protect the measurement area, delaying the effect of edge fracture and rendering rheological measurements at higher shear rates possible. For the shear startup test (Fig. 1C), a step shear rate $\dot{\gamma }$ of various magnitudes is applied to the fluid sample for 300 s. Edge fracture is observed for $\dot{\gamma }\geq 6$ s${}^{-1}$, whose effect is to create an indent that can invade the central 10 mm measurement area of the cone-partitioned plate geometry, causing the measured stress τ to drop abruptly in magnitude. Hence, data obtained after τ has dropped are discarded. The time needed for the indent to invade the measurement area decreases as $\dot{\gamma }$ increases. However, before the invasion event, plateaus of τ can be observed, meaning that the flow can reach a steady state before the indent can invade the measurement area. For the two-step shear test (Fig. 1E), a step shear rate $\dot{\gamma }$ of different magnitudes is first applied to the sample for 1 s, followed by a second step shear rate of 1 s${}^{-1}$ for 300 s. The choice of 1 s for applying the first shear rate ensures that the indent caused by edge fracture will not invade the measurement area during the test. For $\dot{\gamma }\geq 20$ s${}^{-1}$, the flow reaches a steady state before the second step shear rate is applied. The time needed for the flow to become steady decreases as $\dot{\gamma }$ is increased. As the second step shear rate is applied, the shear stress τ converges to the same plateau as that of $\dot{\gamma }=1$ s${}^{-1}$ within around 100 s, indicating that the 180 s of 1 s${}^{-1}$ preshear can indeed eliminate the flow histories of the sample.

For the steady shear test (Fig. 1F), a flow sweep procedure using the parallel plate geometry covered with sandpapers is first employed to obtain the flow curve in a shear rate range of $0.1^{-1}\leq \dot{\gamma }\leq 6$ s${}^{-1}$. For $\dot{\gamma }>6$ s${}^{-1}$, edge fracture occurs, causing the measured stress value to drop. To ensure that the rheological measurement results are independent of the flow configuration, another flow sweep procedure with the cone-partitioned plate geometry is employed to obtain data in the same shear rate range. Subsequently, the steady-state response from the shear startup test is superimposed onto the flow curve in a shear rate range of $1^{-1}\leq \dot{\gamma }\leq 300$ s${}^{-1}$.

The steady shear test fails to measure the first and second normal stress differences $N_{1}$ and $N_{2}$. Such failure is expected in the shear rheometry of yield stress materials; it is caused by the trapped normal stress and drifting of the rheometer’s normal stress signal baseline over time. De Cagny et al. (79) proposed using the large amplitude oscillatory shear test to circumvent such a problem. A certain waveform of the normal stress response could be obtained by applying sinusoidal shear stress of amplitude $\tau_{0}$ and frequency ω to the fluid sample. The normal stress difference could be extracted by measuring the waveform’s amplitude. However, such a method requires a stress-controlled rheometer to be used. The rheometer has to be “hacked” and recalibrated with an oscilloscope, which requires additional expertise and instruments. Also, edge fracture occurs at high shear rates for the TEVP fluid. To measure the normal stresses at those high shear rates, sealing the fluid sample’s free surface using liquid metals (60, 61) or installing a specialized guard-ring (62) is necessary. Hence, the method of De Cagny et al. (79), while ingenious, is considered impractical for the current study. Instead, an ad hoc approach relying on the liquid bridge thinning experiments is employed, the detail of which is described in section “Constitutive modeling.”

### Constitutive modeling

A nonlinear, phenomenological TEVP constitutive model (Eqs. 1 and 2) is employed to describe the rheology of the thermal paste. This model is based on previous models by Varchanis et al. (42), and Stephanou and Georgiou (80). In a previous version of the model (42), the transition from solid to fluid state was based on the von Mises criterion (81, 82), leading to a continuous but abrupt transition between the two states. With the current formalism, the transition is smooth, and no edges are observed in the evolution of the stresses during transient rheometric tests (see Figs. 14–17 in Ref. (42)).

Elasticity is described with the lower convected derivative, which produces a finite second normal stress difference in shear flows. The second normal stress difference has the same magnitude but an opposite sign as the first normal stress difference, i.e. $N_{2}=-N_{1}$. If the more popular upper convected derivative is used instead, the second normal stress difference would be zero, which is inconsistent with the fact that the TEVP fluid undergoes edge fracture in the steady shear test when $\dot{\gamma }>6$ s${}^{-1}$. Other potential choices are the Gordon–Schowalter (83) or Johnson–Segalman (84) convected derivatives, which are essentially linear combinations of the upper and lower convected derivatives. However, these derivatives introduce an extra parameter that ideally should be estimated by normal stress measurements, which are difficult to perform. Assuming that $N_{2}\approx -0.86N_{1}$ to $-0.49N_{1}$ (as suggested in Ref. (79)), the use of the Gordon–Schowalter or Johnson–Segalman derivative will lead to a nonmonotonic flow curve and shear-banding, something that is not observed in the experiment. This would raise the need for a viscous contribution to the total stress tensor, introducing a second additional parameter to the model. The corotational derivative (85), which predicts $N_{2}=-0.5N_{1}$, could be an alternative option in the absence of experimental data for the normal stress differences. However, it again predicts a nonmonotonic flow curve, and a viscous contribution would be necessary. Our intention is not to achieve quantitative agreement with the experiment but to confirm that edge fracture drives the bridge breakup and to obtain deeper insights into the physical mechanism. Thus, we use the simplest possible model to describe the material’s most important rheological fingerprints, leading us to choose the first-order, crude approximation of the lower convected derivative.

All model material parameters are estimated by performing simultaneous nonlinear regression (42) to the SAOS (Fig. 1B), shear startup (Fig. 1D), two-step shear (Fig. 1E), steady shear (Fig. 1F), and liquid bridge thinning experiments (Fig. 6). Specifically, m and n, hence the normal stress differences $N_{1}=-N_{2}$ (red dotted line in Fig. 1F), are tuned such that the simulated liquid bridge radius evolution matches the experimental results at different rotational speeds Ω. This way, while one of the normal stress differences may be under or over-predicted, the one responsible for the liquid bridge deformation process is likely to be correct. As a side note, the creep test results are not used for the nonlinear regression. Nonetheless, the model predicts a yield stress of $\tau_{y}=99$ Pa, which agrees very well with the experimentally measured yield stress $\tau_{y}^{e}=97$ Pa.

It is worth mentioning that Eq. 3 can serve as a quick way to estimate the thixotropic build-up to breakage timescale ratio $k_{1}/k_{2}$. In the frequency sweep experiment (Fig. 1B), the average of $G^{\prime }$ corresponds to the quantity of G(1+m). In the steady shear experiment (Fig. 1F), the magnitude of the shear stress for vanishing shear rates and the slope of the shear stress at high shear rates correspond to $\tau_{y}$ and $\eta_{\;pl}$, respectively. Consequently, it is possible to quantify thixotropic effects without performing transient rheological measurements.

### Simulation setup

The three-dimensional (3D) axisymmetric flow of a TEVP fluid confined between two coaxial circular parallel plates of radius $R_{p}=3$ mm (Fig. 1A) is considered. Initially, the separation distance between the two plates is $H_{0}=0.95$ mm. The liquid bridge is at rest (u=0) in a stress-free condition (τ=0, s=1), forming a cylindrical column between the two plates. Then, the liquid bridge is stretched in the z-direction as the top plate separates from the stationary bottom plate with a velocity $v_{p}=1$ mm s${}^{-1}$. As the separation reaches H=2.5 mm, the liquid bridge is let relaxed for $t_{w}=1$ s. Finally, at t=0 s, the bottom plate accelerates instantaneously to a constant rotational speed Ω, subjecting the liquid bridge to torsion. Regarding the effect of $t_{w}$, see Fig. S1 and supplementary text in the SI Appendix.

Cylindrical coordinates (r,θ,z) are used to describe the problem, where r, θ, and z denote the radial, azimuthal, and axial coordinates, respectively. The fluid velocity is denoted as $u=[u_{r}(r,z),u_{\theta }(r,z),u_{z}(r,z)]$, where each component varies only in the r and z directions because of axisymmetry. Gravity acts in the negative z-direction. The three-phase contact lines formed by the fluid, surrounding air, and solid plates are pinned. The contact angle formed by the fluid and the plates is calculated implicitly from the flow field.

The fluid is of density ρ, and the fluid–air interface is of surface tension σ. The flow is governed by mass and momentum conservations, coupled with the TEVP constitutive model described in Eqs. 1 and 2. The conservation equations for incompressible fluids are:


(5) ∇⋅u=0,

(6) $$\rho \frac{Du}{Dt}=-\nabla P+\nabla \cdot \tau +\rho g,$$

where P is the thermodynamic pressure. Regarding boundary conditions along the free surface, the velocity field satisfies a normal force balance between the surface tension, total stresses in the liquid, and pressure in the surrounding air. The no-slip and no-penetration velocity conditions are imposed on the surfaces of the top and bottom plates. Flow symmetry is imposed on the axis of symmetry. For more detailed descriptions of the boundary conditions, the readers are referred to Varchanis et al. (86). The system of partial differential equations is discretized and solved using the procedure described in Ref. (67). The element size close to the free surface is $h_{\text{e}}\approx 6\times 10^{-5}R_{p}$ and the time step used is dt=0.1/Ω. All simulations are transient and terminate when the neck radius R becomes smaller than 0.01 mm. Regarding the mesh and time step convergence tests, see Fig. S1, Table S1, and supplementary text in the SI Appendix.

## Supplementary Material

pgad042_Supplementary_Data

## Data Availability

All data are included in the manuscript and/or Supplementary Material.
