# Supplementary material for: Edge fracture of thixotropic elastoviscoplastic liquid bridges
Source: PNAS Nexus. 2023 Feb 9;2(3):pgad042. doi: 10.1093/pnasnexus/pgad042 (PMC10011968; doi:10.1093/pnasnexus/pgad042)
Supplement: pgad042_Supplementary_Data [file pgad042_supplementary_data.zip › PNASNEXUS-PNASNEXUS-2022-01158-T-s01.docx]

**
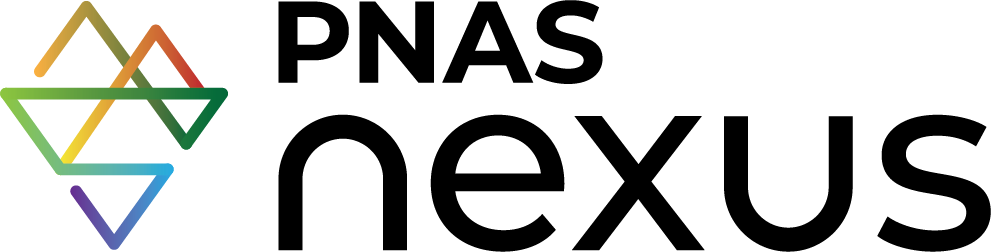
**

**Supplementary Information for**

Edge fracture of thixotropic elastoviscoplastic liquid bridges

Paste the full author list here

San To Chan, Stylianos Varchanis, Amy Q. Shen, and Simon J. Haward

Simon J. Haward
Email: [simon.haward@oist.jp](mailto:simon.haward@oist.jp)

**This PDF file includes:**

Supplementary text

Figure S1

Table S1

Legends for Movies S1 to S3

**Other supplementary materials for this manuscript include the following:**

Movies S1 to S3

**Supplementary Information Text**

**Convergence tests** Figs. S1A and B demonstrate the numerical mesh and time step convergence, respectively. In the mesh convergence study (Fig. S1*A*), we use three consecutively doubled meshes (see Table S1) to probe the spatial accuracy of the numerical scheme. The mesh is highly packed around the midplane of the liquid bridge $z$ = 0.4166$R_{p}$ to ensure high resolution of the free surface curvature and the stresses when the liquid bridge becomes very thin. In the time step convergence study (Fig. S1*B*), we employ three consecutively halved time steps $\delta t$ = 0.2/$\Omega$, 0.1/$\Omega$, and 0.05/$\Omega$ to evaluate the temporal accuracy of the simulations. The simulations are performed for $\Omega$ = 50 rad s^-1^ and the liquid bridge's neck radius $R$ is monitored. The results are mesh- and time step-independent. Mesh M2 and $\delta t$ = 0.1/$\Omega$ are chosen for all simulations. Each simulation takes about 20 minutes when performed in parallel using 128 cores. Finally, we also investigated the effect of the waiting time $t_{w}$ between the initial liquid bridge stretching and the onset of rotation. Fig. S1*C* illustrates that $t_{w}$ = 1 s is enough to guarantee that the liquid bridge thinning by torsion is independent of the initial liquid bridge stretching.


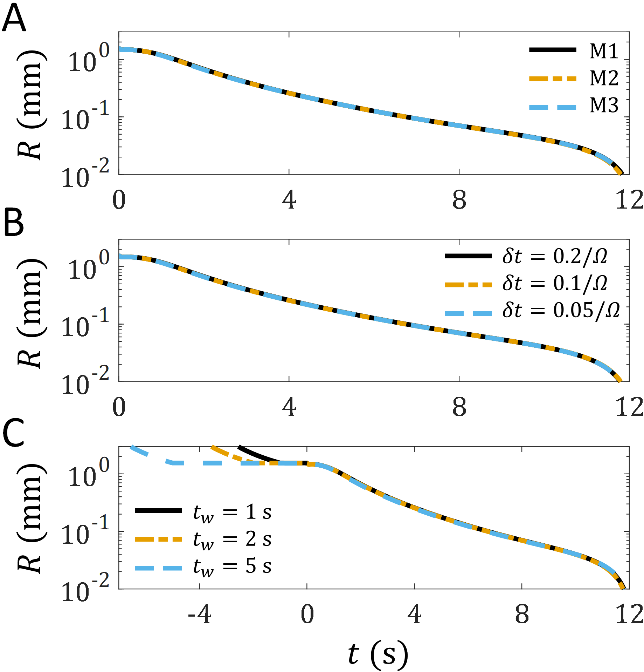


**Fig. S1.** **Convergence tests.** (*A*) Mesh convergence test results showing the simulated neck radius $R$ as a function of time $t$ for different meshes used. For details of the meshes, see Table S1. (*B*) Time step convergence test results showing the simulated neck radius $R$ as a function of time $t$ for different time steps $\delta t$ used. (*C*) The simulated neck radius $R$ as a function of time $t$ for different $t_{w}$, which is the time waited after the initial liquid bridge stretching and before the onset of rotation.

| Mesh | #elements | #nodes | Min. element edge |
| --- | --- | --- | --- |
| M1 | 9720 | 4851 | 8 $\times$ 10^-5^ $R_{p}$ |
| M2 | 38168 | 19065 | 4 $\times$ 10^-5^ $R_{p}$ |
| M3 | 150900 | 75411 | 2 $\times$ 10^-5^ $R_{p}$ |

**Table S1.** Information of the three meshes used in the numerical mesh convergence test.

**Movie S1 (separate file).** Thinning of the TEVP liquid bridge subjected to a rotational speed of $\Omega$ = 20 rad s^-1^.

**Movie S2 (separate file).** Thinning of the TEVP liquid bridge subjected to a rotational speed of $\Omega$ = 50 rad s^-1^.

**Movie S3 (separate file).** Thinning of the TEVP liquid bridge subjected to a rotational speed of $\Omega$ = 150 rad s^-1^.
